# Estimated Annual Deaths, Hospitalizations, and Emergency Department and Physician Office Visits from Foodborne Illness in Ontario

**DOI:** 10.1089/fpd.2018.2545

**Published:** 2019-03-05

**Authors:** Christopher Drudge, Susan Greco, JinHee Kim, Ray Copes

**Affiliations:** ^1^ Public Health Ontario, Toronto, Canada.; ^2^ Dalla Lana School of Public Health, University of Toronto, Toronto, Canada.

**Keywords:** foodborne illness, burden of disease, health care, risk analysis, Ontario, Canada, public health

## Abstract

Public Health Ontario is working to estimate the burden of disease from environmental hazards in Ontario, Canada. As part of this effort, we estimated deaths and health care utilization resulting from exposure to pathogens and toxic substances in food. We applied fractions for the proportion of illness attributable to foodborne transmission to the annual (2008–2012) counts of deaths, hospitalizations, emergency department (ED) visits, and physician office visits for 15 diseases (13 pathogen-specific diseases and 2 nonspecific syndromes) captured by administrative health data. Nonspecific gastroenteritis (causative agent unknown) was the dominant disease, accounting for 98% of ED visits, 94% of hospitalizations, and 88% of deaths annually attributed to the 15 diseases. We estimated that foodborne nonspecific gastroenteritis results in ∼137,000 physician office visits (1000/100,000 population), 40,000 ED visits (310/100,000), 6200 hospitalizations (47/100,000), and 59 deaths (0.45/100,000) in Ontario per year (mean estimates). Our results indicate that pathogen-specific approaches to foodborne disease surveillance can substantially underestimate the deaths and illness resulting from exposure to foodborne pathogens and other causes of foodborne illness.

## Introduction

**FIG. 1.**
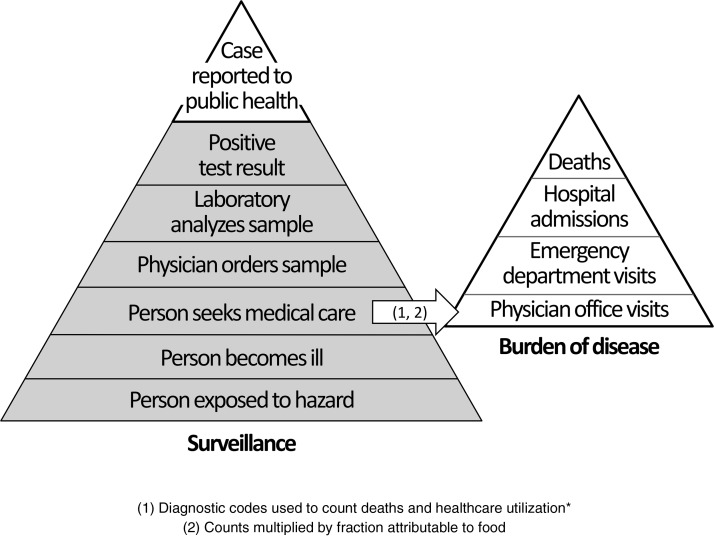
Pyramid model comparing surveillance (left) and burden of disease (right) approaches. Surveillance pyramid image adapted with permission from Centers for Disease Control and Prevention (2015). *May or may not be laboratory confirmed.

Understanding the public health risks associated with consuming foods containing pathogens and toxic substances can help focus efforts to reduce foodborne illness. The burden, or adverse health impact, resulting from foodborne illness can be envisioned as a pyramid: many people experience mild illness, fewer seek medical care, and even fewer are hospitalized or die (Fig. 1). There are challenges in estimating the burden of foodborne illness at all tiers of the pyramid, but illnesses serious enough to require medical care are of particular interest to decision makers in Ontario, Canada (∼13.4 million people in 2016), as health care is publicly funded.

One approach to estimating the burden of foodborne illness is to use data derived from appropriately designed surveillance systems. In Ontario, surveillance data are collected for a limited group of diseases of public health significance (Ontario Ministry of Health and Long-Term Care, 2018a). However, the source of exposure (e.g., food and water) is often unclear or unknown (Vrbova *et al.*, 2012), as is the level of medical care (e.g., physician office visit vs. hospitalization) used in treatment of the illness. Ontario reportable disease surveillance data (sporadic and outbreak cases) are mostly collected from provincial laboratories, which do not capture cases for which samples are not ordered and submitted for laboratory culture.

Another approach to estimating foodborne illness burden is modeling, whereby a fraction of mortality and morbidity measures (e.g., deaths and disability-adjusted life years [DALYs]) are attributed to foodborne agents. The World Health Organization estimated the global burden of foodborne diseases for 31 pathogens, toxins, and chemicals (World Health Organization, 2015). However, many of the diseases were not important in Canada (e.g., *Vibrio cholerae*), and results were presented in terms of cases of illness, which lack information on illness severity, and DALYs, which are excellent for international comparisons but can lack resonance with local policy makers. Two recent modeling studies estimated the burden of foodborne illness in Canada for 30 specific pathogens and unspecified agents (Thomas *et al.*, 2013, 2015). These studies have been cited by food safety materials produced at the federal (Government of Canada, 2016) and provincial (Public Health Ontario, 2014) levels in Canada. However, the burden did not include estimates of emergency department (ED) visits or physician office visits, which are expected to be substantial because of the mild-to-moderate nature of most foodborne illness. Ontario-specific burden estimates are also lacking for sequelae of foodborne infections, and updated estimates based on more recent foodborne attribution estimates and more recent and Ontario-specific outcome data are needed.

Public Health Ontario is working to estimate the burden of disease resulting from exposure to many groups of environmental hazards, with the goal of informing decision makers about the relative contributions of each. In this study, we estimated the burden of disease from one of these hazard groups, pathogens and toxic substances in food. Through our analysis, we addressed knowledge gaps concerning the burden of foodborne illness (defined for this study as deaths and health care utilization resulting from cases of foodborne illness) in Ontario.

## Materials and Methods

For 15 hazard-disease pairings (e.g., listeriosis caused by *Listeria monocytogenes*), we calculated a burden estimate by multiplying the annual count of deaths and instances of health care utilization (i.e., hospitalizations, ED visits, and physician office visits) by attributable fractions (AFs) describing the proportion attributable to all food. Our approach parallels that of the World Health Organization in a recent global environmental burden of disease study (Prüss-Ustün *et al.*, 2016). For the World Health Organization study, burden was estimated for a disease by multiplying disease outcomes (deaths and DALYs) by an AF calculated using one of several approaches, including expert survey (Prüss-Ustün *et al.*, 2016). We performed the analysis using @RISK, a risk analysis add-in for Microsoft Excel (version 7.5; Palisade Corporation), which permitted us to incorporate the uncertainty associated with AFs and variability in annual outcome counts by defining these input parameters as probability distributions. Burden estimates were produced using Latin Hypercube sampling (10,000 iterations) (McKay *et al.*, 1979). Final estimates were summarized using a mean and 90% prediction interval (PI). We reported estimates for pairings that annually caused at least 10 ED visits, 10 hospitalizations, or 1 death (hereafter referred to as the threshold for reporting). To check for differences in outcomes across age and sex, we conducted a Poisson regression analysis on the death and health care utilization counts. See Age and Sex Analysis for Nonspecific Gastroenteritis section of the Supplementary Data for details of this analysis (Supplementary Data are available online at www.liebertpub.com/fpd).

### Selection of hazard-disease pairings

To capture the top contributors to foodborne illness in Ontario, we focused on a group of 15 hazard-disease pairings, 13 of which were specific to 11 pathogens (Table 1). We identified pathogens expected to be important contributors by examining two data sources: (1) provincial surveillance data (Public Health Ontario, 2017) and (2) a recent Canadian study reporting annual hospitalizations and deaths for 30 foodborne pathogens (Thomas *et al.*, 2015). See Materials and Methods—Selection of Hazard-Disease Pairings section of the Supplementary Data for further details. For the hazards *Campylobacter* spp. and verotoxin-producing *Escherichia coli* (VTEC), in addition to enteric disease, we included *Campylobacter*-associated Guillain-Barré syndrome (GBS) and VTEC-associated hemolytic-uremic syndrome (HUS) because of their expected major contribution to each pathogen-specific burden (Ruzante *et al.*, 2011). We also included two nonspecific syndromes, food poisoning caused by various bacterial toxins and nonspecific gastroenteritis from pathogens, toxins, and other harmful substances. The nonspecific syndromes were included based on a literature review of burden of disease studies conducted in high-income countries and published since 2008.

**Table 1.** Foodborne Hazard-Disease Pairings and Associated Foodborne Attributable Fractions and Disease Diagnostic Codes

*Hazard and disease*	*Foodborne AF (%)*		*Diagnostic code(s)*	
	*Mean*	*90% PI*	*ICD-10/ICD-10-CA code(s)*	*OHIP diagnostic code*^a^
Specific pathogens				
*Campylobacter* spp. intestinal infection (campylobacteriosis)	49	32–65	A04.5	
*Campylobacter*-associated GBS	49	32–65	G61.0^b^	
*Cryptosporidium* spp. intestinal infection (cryptosporidiosis)	14	3–29	A07.2	
*Giardia* spp. intestinal infection (giardiasis)	9	2–17	A07.1	
Hepatitis A virus infection (acute)	30	9–52	B15.x	
*Listeria monocytogenes* infection (listeriosis)	58	43–74	A32.x	
Norovirus intestinal infection	19	5–34	A08.1	
*Salmonella* spp. (nontyphoidal) infection (salmonellosis)	46	29–63	A02.x	
*Shigella* spp. intestinal infection (shigellosis)	24	9–40	A03.x	
*Toxoplasma gondii* infection (toxoplasmosis)	42	16–66	B58.x	
VTEC intestinal infection	55	41–70	A04.3	
VTEC-associated HUS	55	41–70	D59.3^b^	
*Yersinia enterocolitica* intestinal infection (yersiniosis)	78	68–88	A04.6	
Nonspecific syndromes				
Food poisoning	100	None (constant)	A05.x	005
Nonspecific gastroenteritis	39	14–65	A04.9, A07.9, A08.4, A09^c^, A09.0, A09.9^d^, K52.9^d^, R19.x^d^	009^d^

^a^ Due to the limited number of pathogen-specific OHIP diagnostic codes, we were unable to estimate total pathogen-specific physician office visits.

^b^ Diagnostic code captured all causes of the syndrome, so we estimated the proportion of illnesses due to the specified pathogen for each year. We used a range of 0.09–0.47 (as a uniform distribution) for *Campylobacter*-associated GBS and a range of 0.75–1.00 (as a uniform distribution) for VTEC-associated HUS (see Guillain-Barré Syndrome and Hemolytic-Uremic Syndrome section of the Supplementary Data for further details).

^c^ In April 2009, the ICD-10/ICD-10-CA code A09 (diarrhea and gastroenteritis of presumed infectious origin) was replaced by A09.0 (other and unspecified gastroenteritis and colitis of infectious origin) and A09.9 (gastroenteritis and colitis of unspecified origin).

^d^ These diagnostic codes captured unspecified gastroenteritis, so we estimated the proportion due to pathogens or toxic substances (i.e., potential foodborne agents), as opposed to preexisting conditions, for each year. We used a range of 0.81–0.88 (as a uniform distribution) (see Nonspecific Gastroenteritis section of the Supplementary Data for further details).

AF, attributable fraction; GBS, Guillain-Barré syndrome; HUS, hemolytic-uremic syndrome; ICD, International Classification of Disease; OHIP, Ontario Health Insurance Plan; PI, prediction interval; VTEC, verotoxin-producing *Escherichia coli*.

### Identification of AFs

We developed AFs for each hazard-disease pairing (Table 1) using estimates from the indexed scientific literature. We derived AFs for all 11 pathogens from one recent Canadian expert elicitation study that reported probability distributions of attribution for major transmission routes (foodborne, waterborne, animal contact, person-to-person, and other) for domestically-acquired enteric pathogens (Butler *et al.*, 2015). For each pathogen, the foodborne AF distribution was calculated as 100% minus the sum of the AF distributions for all other transmission routes. For VTEC, we used the foodborne probability distribution reported for VTEC O157 because we expected most VTEC infections in Ontario to be this serotype based on past surveillance data (Public Health Ontario, 2017). For nonspecific gastroenteritis, we created an AF from literature-sourced Canadian estimates describing the proportion of gastroenteritis attributable to food (Keegan *et al.*, 2009; Vrbova *et al.*, 2012; Thomas *et al.*, 2013; Lukacsovics *et al.*, 2014; Whitfield *et al.*, 2017). See Materials and Methods—Identification of Attributable Fractions section of the Supplementary Data for further details. We used the distribution-fitting tool in @RISK to determine the best-fitting AF distribution for each hazard-disease pairing (based on Akaike information criterion value) (Palisade, 2016). Distribution parameters for foodborne AFs are provided in Supplementary Table S1.

### Analysis of health outcome data

Comprehensive data are available for health outcomes indicative of moderate-to-severe illness in Ontario. We acquired data for 2008–2012 from IntelliHealth Ontario, an online repository maintained by the Ontario Ministry of Health and Long-Term Care (Ontario Ministry of Health and Long-Term Care, 2018b).

For each hazard-disease pairing, we counted deaths, hospitalizations, and ED visits using diagnostic codes for administrative health databases (outcomes assigned pathogen-specific codes may or may not be laboratory confirmed) (Table 1). Due to the limited number of pathogen-specific diagnostic codes assigned to physician office visits in Ontario, we were only able to capture physician office visits for 2 of the 15 pairings: food poisoning and gastroenteritis. The annual counts were treated as a discrete distribution with 20% of the probability mass at the count for each individual year. We used the same approach as for counts to describe death and health care utilization rates, which we calculated using the Ontario population for each year from 2008 to 2012. See Materials and Methods—Analysis of Health Outcome Data section of the Supplementary Data for further details.

Most cases of foodborne illness occur within days or weeks of exposure, so we assumed that deaths and health care utilization were proximate to exposure. We did not attempt to exclude travel-related illness for each pairing, as it still contributes to illness in Ontario residents. We also did not adjust for under-ascertainment associated with laboratory testing and capture by administrative health databases, as we wished to generate burden estimates comparable across the foodborne illness hazard-disease pairings in this study. Annual counts for unattributed health outcomes are provided in Supplementary Table S2.

## Results

As a percentage of mean annual outcomes for the 15 hazard-disease pairings included in our analysis, the nonspecific syndromes were dominant. For the pairings included in our analysis, nonspecific gastroenteritis accounted for the vast majority of ED visits (98%), hospitalizations (94%), and deaths (88%) attributed to foodborne agents.

We estimated that the burden of nonspecific gastroenteritis in Ontario from exposure to pathogens and toxic substances in food was ∼137,000 (90% PI 47,000–227,000) physician office visits, 40,000 (90% PI 14,000–67,000) ED visits, 6200 (90% PI 2100–10,300) hospitalizations, and 59 (90% PI 20–103) deaths per year (Table 2). For the nonspecific syndromes (gastroenteritis and food poisoning), the mean annual count of physician office visits was at least 3-fold higher than ED visits, ED visits at least 6-fold higher than hospitalizations, and hospitalizations at least a 100-fold higher than deaths (Table 2). However, this pattern was not observed for any of the pathogen-specific diseases or their sum. For the sum of specific pathogens, the mean annual count of ED visits was close to hospitalizations, and both were about 40-fold higher than deaths.

**Table 2.** Estimated Annual Deaths and Health Care Utilization (Counts and Rates/100,000 Population) Attributed to Select Foodborne Pathogen Hazard-Disease Pairings in Ontario

*Hazard and disease*^a,b^	*ED visits*	*Hospitalizations*	*Deaths*
	*Mean (90% PI)*	*Mean (90% PI)*	*Mean (90% PI)*
Counts			
Nonspecific gastroenteritis	40,000 (14,000–67,000)	6200 (2100–10,300)	59 (20–103)
Food poisoning	550 (360–750)	42 (33–48)	<1
*Salmonella* spp. (nontyphoidal) infection	110 (70–150)	140 (90–200)	1 (0–3)
*Campylobacter* spp. intestinal infection	90 (60–120)	90 (50–130)	<1
*Campylobacter*-associated GBS	25 (9–49)	35 (13–62)	1 (0–2)
*Listeria monocytogenes* infection	19 (3–77)	28 (18–48)	3 (1–8)
Hepatitis A virus infection	16 (4–30)	14 (4–26)	<1
Norovirus intestinal infection	12 (2–35)	24 (5–61)	1 (0–3)
VTEC-associated HUS	<10	23 (15–33)	<1
Other specific pathogens	24 (17–33)	31 (21–40)	<1
All specific pathogens	300 (230–390)	380 (300–470)	8 (4–13)
All pairings	41,000 (15,000–68,000)	6600 (2600–10,700)	67 (28–110)
Rates (per 100,000 population)			
Nonspecific gastroenteritis	310 (110–520)	47 (16–79)	0.45 (0.15–0.78)
Food poisoning	4.2 (2.6–5.7)	0.32 (0.25–0.37)	<0.01
*Salmonella* spp. (nontyphoidal) infection	0.84 (0.53–1.18)	1.1 (0.7–1.5)	<0.01
*Campylobacter* spp. intestinal infection	0.69 (0.44–0.94)	0.66 (0.41–0.95)	<0.01
*Campylobacter*-associated GBS	0.19 (0.07–0.37)	0.26 (0.10–0.48)	<0.01
*Listeria monocytogenes* infection	0.15 (0.03–0.59)	0.22 (0.13–0.37)	0.025 (0.007–0.065)
Hepatitis A virus infection	0.12 (0.03–0.23)	0.11 (0.03–0.20)	<0.01
Norovirus intestinal infection	<0.1	0.19 (0.04–0.46)	<0.01
VTEC-associated HUS	<0.1	0.17 (0.11–0.25)	<0.01
Other specific pathogens	0.19 (0.13–0.25)	0.23 (0.16–0.31)	<0.01
All specific pathogens	2.3 (1.8–3.0)	2.9 (2.3–3.6)	0.058 (0.028–0.102)
All pairings	310 (110–520)	50 (20–82)	0.51 (0.21–0.84)

^a^ We reported results for the 7 pathogen-specific pairings responsible for at least 10 ED visits, 10 hospitalizations, or 1 death per year (mean count). The remaining six pathogen-specific pairings are summarized as other specific pathogens. Results may not add up due to rounding (to two significant figures for results above reporting thresholds).

^b^ Foodborne nonspecific gastroenteritis and food poisoning were responsible for 137,000 (90% PI 47,000–227,000) and 2200 (90% PI 2000–2400) physician office visits, respectively (mean annual estimates). The corresponding physician office visit rates per 100,000 population were 1000 (90% PI 400–1700) and 16 (90% PI 15–18), respectively (mean annual estimates).

ED, emergency department; GBS, Guillain-Barré syndrome; HUS, hemolytic-uremic syndrome; PI, prediction interval; VTEC, verotoxin-producing *Escherichia coli*.

Among pathogen-specific pairings, the estimated mean annual ED visits and hospitalizations were highest for nontyphoidal *Salmonella* spp. and *Campylobacter* spp. (Table 2). *Campylobacter*-associated GBS accounted for 22% of ED visits and 29% of hospitalizations for foodborne *Campylobacter* spp. infections. Of the 13 pathogen-specific pairings we considered, 7 met our threshold for reporting.

## Discussion

Our results indicate that the majority of foodborne deaths, hospitalizations, and ED visits in Ontario are associated with nonspecific gastroenteritis rather than a specific pathogen. Underestimation of these outcomes is likely a consequence of the multiple steps necessary for a case of pathogen-specific illness to be captured by public health surveillance (Fig. 1). For example, a recent Canada-wide telephone survey revealed that only 9% of people experiencing an episode of acute gastrointestinal illness sought medical care, of which less than one in five was asked to submit a sample for laboratory testing (Thomas *et al.*, 2017). The lack of an identified pathogen for most deaths, hospitalizations, and ED visits suggests that pathogen-specific approaches to infectious disease surveillance can substantially underestimate the burden of disease due to food. For physician office visits attributed to foodborne agents, the limited number of pathogen-specific diagnostic codes prevented us from estimating the proportion of visits associated with nonspecific gastroenteritis rather than a specific identified pathogen. Our inability to capture physician office visits due to specific foodborne pathogens highlights an important gap in Ontario administrative health data.

Our finding that nonspecific gastroenteritis rather than specific pathogens was responsible for the majority of foodborne illness burden in Ontario is consistent with other Canadian burden studies. Even after accounting for underreporting and underdiagnosis, Thomas *et al.* (2013, 2015) found that illness related to unspecified foodborne agents exceeded illness from 30 specific pathogens for cases of illness, hospitalizations, and deaths. In looking at hospitalization due to acute gastrointestinal illness (not specifically attributed to foodborne exposures), Fleury *et al.* (2008) found that the majority (78%) of hospitalizations had no specific pathogen identified. The major contribution of unidentified etiologic agents to foodborne illness has also been reported for other high-income countries (Scallan *et al.*, 2011; Kirk *et al.*, 2014).

Our annual estimates for nonspecific syndromes followed the burden of illness pyramid model with respect to expected illness severity (physician office visits > ED visits > hospitalizations > deaths). Our burden estimates did not include mild illnesses for which medical care was not sought, and so should be viewed as underestimates of true burden. Accordingly, our annual estimate for physician office visits due to nonspecific gastroenteritis was two orders of magnitude lower than the ∼12 million episodes of acute gastrointestinal illness estimated to occur in Ontario every year (Sargeant *et al.*, 2008). We did not observe the same pattern as the nonspecific syndromes for the sum of specific pathogens, as the mean annual count of ED visits was close to hospitalizations and both were higher than deaths. This difference prevented us from estimating pathogen-specific physician office visits using the ratio of ED visits to physician office visits for nonspecific syndromes.

We estimated *Campylobacter* spp. and nontyphoidal *Salmonella* spp. as the top two causes of pathogen-specific ED visits and hospitalizations, in agreement with contemporaneous results reported for laboratory-confirmed cases of enteric diseases under surveillance in Ontario (Vrbova *et al.*, 2012; Public Health Agency of Canada, 2017) (Table 2). These two pathogens also ranked among the top four pathogen-specific causes of domestic foodborne illness-related hospitalizations in Canada, as estimated using administrative health data, surveillance data, and literature estimates (Thomas *et al.*, 2015). In contrast to our findings, two recent Canadian studies identified norovirus as the top contributor to pathogen-specific foodborne illness-related cases and hospitalizations (Thomas *et al.*, 2013, 2015). The authors based their estimate on the proportion of nonspecific acute gastrointestinal illness thought to be due to the virus. Our burden estimation approach was instead dependent on the identification of norovirus, likely by laboratory confirmation, as the cause of an illness, which explains our comparatively low estimates for this disease. Others have reported that norovirus is likely responsible for an appreciable proportion of nonspecific gastroenteritis captured by administrative health data (Van Asten *et al.*, 2011). We identified GBS as a major contributor to the burden associated with foodborne *Campylobacter* spp., and our inclusion of this disease is novel for a foodborne illness burden study in Canada. Although *Campylobacter* infections are reportable in Ontario, *Campylobacter*-associated GBS is not reportable as a specific complication.

This study has several limitations. The AFs we developed were based on episodes of illness, not deaths or health care utilization. However, most but not all of the pathogens we examined tend to cause acute illness, so a single episode of illness, rather than multiple bouts of illness or chronic illness, is the usual outcome. The AFs for specific pathogens were based on an expert elicitation involving 31 experts (Butler *et al.*, 2015), which may not be representative of opinions among all experts in the larger scientific community. However, the expert elicitation was specific to Canada, and our use of probabilistic modeling allowed us to reflect the range in expert attribution to food. The AF for nonspecific gastroenteritis was based on studies reporting the proportion of foodborne illness cases for known pathogens, which may not be representative of foodborne illness from unknown causes. However, the range is consistent with the pathogen-specific pairings. We estimated physician office visits using aggregate data and were unable to estimate total visits due to specific pathogens because of the limited number of diagnostic codes used to describe these visits. We selected AFs applicable to Ontario, so others wanting to replicate our approach in other countries should use locally appropriate AFs, as well as local information on morbidity and mortality. This can address cross-national differences in access to health care, population demographics, and risk of foodborne illness. Finally, our burden estimate did not capture episodes of illness for which medical care was not sought. Nevertheless, by restricting our analysis to deaths and health care utilization, we assume that we have captured most moderate-to-severe illness due to foodborne causes and are able to compare these results to the burden of other environmental hazards in Ontario.

This study also has a number of strengths and novel findings with respect to foodborne illness in Ontario. First, our use of comprehensive and often high quality administrative health data allowed us to generate robust estimates of deaths and health care utilization. In particular, we were able to generate estimates of ED visits and physician office visits, outcomes not included in previous provincial burden studies. These estimates can be of use to provincial decision makers, as well as others who could adopt our approach for their jurisdiction. Second, we estimated the burden of several important diseases not under surveillance in Ontario (e.g., *Campylobacter*-associated GBS). Third, our estimates reflect uncertainty in foodborne AFs, as well as year-to-year variation in counts of deaths and health care utilization. Thus, the plausible range we report is a more credible estimate than point estimates that do not capture any of the underlying uncertainty and variability in the inputs.

A public health surveillance system that focuses on syndromes (e.g., nonspecific gastroenteritis) may better capture the true burden of foodborne illness, whether it is in terms of cases, health care utilization, or deaths. Syndrome-based surveillance approaches such as telephone surveys (Thomas *et al.*, 2017) and social media data mining (Harris *et al.*, 2014), which bypass the complex reporting infrastructure required to capture pathogen-specific illnesses, are likely also capable of providing more rapid estimates of foodborne illness burden.

## Conclusions

We identified nonspecific gastroenteritis as the major contributor to the burden of foodborne illness in Ontario. Nonspecific gastroenteritis is not well captured by current public health surveillance efforts in Ontario. Future steps to include nonspecific gastroenteritis, alongside efforts to better capture pathogen-specific surveillance data, represent an opportunity to improve food safety. We anticipate that the approach outlined in this article will inform future foodborne illness burden estimation efforts in Ontario, as well as burden estimation efforts in other jurisdictions, with the ultimate goal of facilitating initiatives aimed at reducing foodborne illness.

## Supplementary Material

Supplemental data
