# Supplementary material for: Estimated Annual Deaths, Hospitalizations, and Emergency Department and Physician Office Visits from Foodborne Illness in Ontario
Source: Foodborne Pathog Dis. 2019 Mar 5;16(3):173–9. doi: 10.1089/fpd.2018.2545 (PMC6434595; doi:10.1089/fpd.2018.2545)
Supplement: Supplemental data [file Supp_Data.pdf]

Supplementary Data

Materials and Methods—Selection of Hazard-Disease Pairings

To facilitate our analysis, we identified pathogens expected to be important contributors to the foodborne pathogen burden in Ontario by compiling and ranking two separate sets of disease incidence measures for pathogens known to cause foodborne illness. For each set, we multiplied the overall incidence by the fraction attributable to foodborne transmission (Butler *et al.*, 2015). The first set of estimates was based on the mean annual Ontario incidence rate (calendar years 2006–2015) for foodborne illness-associated pathogens captured by Ontario public health surveillance data (i.e., integrated Public Health Information System (iPHIS) data, which consist largely of laboratory-identified cases of reportable diseases) (Public Health Ontario, 2017). The second set of estimates was based on the sum of mean annual reported deaths and hospitalizations in Canada (years 2000–2010 or 2007–2010, depending on the pathogen) for a comprehensive list of foodborne illness-associated pathogens (Thomas *et al.*, 2015). We excluded pathogens not known to be transmitted in Ontario (e.g., *Salmonella enterica* serotype Typhi). There was good concordance between both sets of estimates. We selected the top 10 pathogens from each ranked set to include in our burden estimation. As there was substantial overlap between the ranked lists, we ended up with 11 pathogens. A similar rank-based selection process has been used by other foodborne illness burden studies to identify the most important pathogens (e.g., Hoffmann *et al.*, 2012). Notably, other pathogens may be important for certain populations [e.g., brucellosis and trichinosis in indigenous peoples (Jung and Skinner, 2017)].

For the first of the two ranked sets of incidence estimates used to select hazard-disease pairings, iPHIS data were either not available or did not capture the general Ontario population for the following pathogens: adenovirus, norovirus, *Vibrio parahaemolyticus*, *Vibrio vulnificus*, and other *Vibrio* spp. (excluding *Vibrio cholerae*). To address this deficiency, we identified additional Canadian annual incidence rates. We found one estimate for the *Vibrio* groups (Thomas *et al.*, 2013), two estimates for adenovirus (Public Health Agency of Canada, 2012; Thomas *et al.*, 2013), and three estimates for norovirus (Public Health Agency of Canada, 2012; Thomas *et al.*, 2013; Morton *et al.*, 2015). For each pathogen, we used all available estimates to calculate a mean annual incidence rate, which was used in the iPHIS ranked set of estimates.

Materials and Methods—Identification of Attributable Fractions

Attributable fractions (AFs) describe the proportion of disease outcomes (e.g., hospitalizations due to norovirus infection) that are foodborne. More precisely, the AFs represent the proportional reduction in disease that would occur if exposures through food were eliminated (Prüss-Ustün *et al.*, 2016). Given the geographic variation of foodborne illness risk factors, we sought to use AFs that were as representative of Ontario as possible. Distribution parameters for foodborne AFs are provided in Supplementary Table S1.

To develop an AF for nonspecific gastroenteritis (infectious or unspecified), we identified literature-sourced estimates of the proportion of illness attributable to food. Studies were identified by a review of all indexed scientific literature for research conducted in Canada and published in the last 10 years (2008–2018). We found five studies reporting estimates of gastroenteritis attributable to food (Keegan *et al.*, 2009; Vrbova *et al.*, 2012; Thomas *et al.*, 2013; Lukacsovics *et al.*, 2014; Whitfield *et al.*, 2017). These estimates, which were either point values or probability distributions, were composites based on selected groups of known causes for which surveillance data or source attribution expert elicitations were available. The best-fitting distribution for the five estimates (point value or central value of distribution) was determined using the Microsoft Excel add-in @RISK (version 7.5; Palisade Corporation).

Materials and Methods—Analysis of Health Outcome Data

Data sources and diagnostic codes

Vital statistics mortality data are collected from death certificates by the Ontario Office of the Registrar General. The Discharge Abstract Database (DAD) and National Ambulatory Care Reporting System (NACRS) capture hospitalizations and ambulatory care visits and are products of the Canadian Institute for Health Information (CIHI). The Ontario Health Insurance Plan (OHIP) database captures services billed through OHIP by health care providers in Ontario and is administered by the Ontario Ministry of Health and Long-Term Care (MOHLTC). The MOHLTC compiles the four data sources and makes them available through an online repository (IntelliHealth Ontario). Reabstraction studies by CIHI have identified DAD and NACRS as accurate and comprehensive sources of Ontario hospitalization and emergency department (ED) visit data (CIHI, 2008, 2012). The OHIP database covers all payments made by the MOHLTC for services insured by OHIP and billed by health care providers (primarily physicians), which represent the vast majority of physician services provided in Ontario. Annual counts for unattributed health outcomes are provided in Supplementary Table S2.

For deaths, hospitalizations, and ED visits, record-level data were extracted using diagnostic codes representing each hazard-disease pairing (e.g., listeriosis caused by *Listeria monocytogenes*). Diagnostic codes adhered to the *International Statistical Classification of Diseases, 10th Revision* (ICD-10). While deaths were described using ICD-10, diagnoses associated with a hospitalization or ED visit followed the Canadian version of the coding standard (ICD-10-CA). Most diseases were clearly captured by a single diagnostic code (e.g., A07.1 for giardiasis). We considered the diagnosis type associated with each diagnostic code in DAD and NACRS to decide what code best represented a hospitalization or ED visit. Following other burden of disease estimates for Ontario (Kwong *et al.*, 2010), we estimated deaths using the single underlying cause. For physician office visits, aggregate data were extracted using diagnostic codes specific to

Supplementary Table S1. Distribution Parameters for Foodborne Attributable Fractions (%)

Hazard and disease	*Distribution*[a]	*Parameters (%)*
Campylobacter spp. intestinal infection (campylobacteriosis)	Normal	Mean, standard deviation: 48.67, 9.87
Campylobacter-associated GBS	Normal	Mean, standard deviation: 48.67, 9.87
Cryptosporidium spp. intestinal infection (cryptosporidiosis)	Beta	Alpha1, alpha2, minimum, maximum: 2.31, 6.72, –0.87, 57.41
Giardia spp. intestinal infection (giardiasis)	Beta	Alpha1, alpha2, minimum, maximum: 2.92, 9.91, –0.97, 40.74
Hepatitis A virus infection (acute)	Beta	Alpha1, alpha2, minimum, maximum: 5.52, 7.71, –12.20, 88.26
Listeria monocytogenes infection (listeriosis)	Normal	Mean, standard deviation: 58.27, 9.39
Norovirus intestinal infection	Beta	Alpha1, alpha2, minimum, maximum: 4.55, 8.58, –5.15, 63.30
Salmonella spp. (nontyphoidal) infection (salmonellosis)	Beta	Alpha1, alpha2, minimum, maximum: 19.54, 30.55, –12.28, 136.37
Shigella spp. intestinal infection (shigellosis)	Beta	Alpha1, alpha2, minimum, maximum: 9.32, 16.34, –13.27, 89.39
Toxoplasma gondii infection (toxoplasmosis)	Beta	Alpha1, alpha2, minimum, maximum: 18.71, 11.25, –68.65, 108.46
VTEC intestinal infection	Beta	Alpha1, alpha2, minimum, maximum: 13.13, 16.22, 12.22, 108.95
VTEC-associated HUS	Beta	Alpha1, alpha2, minimum, maximum: 13.13, 16.22, 12.22, 108.95
Yersinia enterocolitica intestinal infection (yersiniosis)	Beta	Alpha1, alpha2, minimum, maximum: 10.65, 10.60, 48.98, 106.15
Food poisoning	Constant	100
Nonspecific gastroenteritis	Uniform	Minimum, maximum: 10.81, 67.68

[a]Distributions were truncated at 0% and 100%.

GBS, Guillain-Barré syndrome; HUS, hemolytic-uremic syndrome; VTEC, verotoxin-producing *Escherichia coli*.

the OHIP database. Due to the limited availability of pathogen-specific diagnostic codes, we only captured visits for the two nonspecific syndrome pairings: food poisoning (diagnostic code 005) and gastroenteritis (diagnostic code 009). If a case of illness required multiple contacts with the health care system, each contact was counted as an instance of health care utilization.

A hospitalization (DAD) or ambulatory care visit (NACRS) could be assigned multiple ICD-10-CA codes. To ensure that only illnesses that substantially contributed to a hospitalization or ED visit were captured in our burden estimate, we considered only a limited group of diagnoses based on their assigned type (DAD: most responsible diagnosis, preadmit comorbidity, postadmit comorbidity, or service transfer diagnosis; NACRS: main problem or other problem). Query diagnoses, which indicated that the health care provider had documented uncertainty in the diagnosis, were also excluded from our analysis. Each hospitalization or ambulatory care visit had a unique identifier, permitting all of the diagnoses associated with it to be grouped together. To estimate ED visits from NACRS data, we excluded ambulatory care visit types other than unscheduled ED visits. For hospitalizations and ED visits, the calendar year was based on the discharge date.

In accordance with Ontario standards, we defined verotoxin-producing *Escherichia coli* (VTEC) using the ICD-10/ICD-10-CA code A04.3 (enterohemorrhagic *E. coli* infection) (Ontario Ministry of Health and Long-Term Care, 2014). For food poisoning, we used all subcategories of the ICD-10/ICD-10-CA code A05 (other bacterial foodborne intoxications, not elsewhere classified). Although this group of diagnostic codes excluded *E. coli* infection, listeriosis, and salmonellosis, each of these diseases was separately included as pathogen-specific hazard-disease pairings in our burden estimation approach.

Guillain-Barré syndrome and hemolytic-uremic syndrome

The ICD-10/ICD-10-CA codes for Guillain-Barré syndrome (GBS) (G61.0) and hemolytic-uremic syndrome (HUS) (D59.3) captured all causes of these illnesses. Therefore, we used literature sources to estimate the proportion of GBS and HUS deaths, hospitalizations, and ED visits associated with *Campylobacter* and VTEC infections, respectively. For both GBS and HUS, studies were identified by a review of all indexed scientific literature for research conducted in high-income countries in North America, Europe, or Australasia and published in the last 20 years (1998–2018). The proportion for GBS was based on seven studies (Guarino *et al.*, 1998; Jacobs *et al.*, 1998; Hadden *et al.*, 2001; Koga *et al.*, 2001; Drenthen *et al.*, 2011; Sivadon-Tardy *et al.*, 2014; Zautner *et al.*, 2014) reporting the proportion of GBS cases for which there was serological evidence of a recent *Campylobacter* infection. The proportion for HUS was based on eight studies (Elliott *et al.*, 2001; Gerber *et al.*, 2002; Tozzi *et al.*, 2003; Constantinescu *et al.*, 2004; Lynn *et al.*, 2005; Espié *et al.*, 2008; Ardissino *et al.*, 2016; Jenssen *et al.*,

Supplementary Table S2. Annual Counts for Unattributed Health Outcomes in Ontario, 2008–2012

Hazard and disease	*Annual count (minimum/mean/maximum)*[a]		
	ED visits	*Hospitalizations*	*Deaths*
Campylobacter spp. intestinal infection (campylobacteriosis)	167/185/204	151/178/227	0/0/2
Campylobacter-associated GBS	37/52/63	64/71/78	1/2/3
Cryptosporidium spp. intestinal infection (cryptosporidiosis)	10/17/22	6/10/15	0/0/0
Giardia spp. intestinal infection (giardiasis)	40/56/74	17/21/26	0/0/0
Hepatitis A virus infection (acute)	37/52/63	37/47/62	0/1/1
Listeria monocytogenes infection (listeriosis)	7/33/118	37/49/74	2/6/13
Norovirus intestinal infection	26/62/143	67/132/247	2/7/11
Salmonella spp. (nontyphoidal) infection (salmonellosis)	214/241/262	271/307/362	1/3/5
Shigella spp. intestinal infection (shigellosis)	16/21/24	21/24/26	0/0/0
Toxoplasma gondii infection (toxoplasmosis)	8/10/14	15/21/26	0/0/1
VTEC intestinal infection	0/2/5	7/10/16	0/0/0
VTEC-associated HUS	15/16/16	33/41/53	0/1/2
Yersinia enterocolitica intestinal infection (yersiniosis)	6/8/11	4/9/15	0/0/0
Food poisoning	355/550/751	33/42/48	0/0/1
Nonspecific gastroenteritis	95,989/103,211/109,341	14,658/15,737/16,470	128/151/171

[a]Annual physician office visits in Ontario (minimum/mean/maximum) for food poisoning were 1972/2163/2392 and for nonspecific gastroenteritis were 336,090/349,166/359,603.

ED, emergency department; GBS, Guillain-Barré syndrome; HUS, hemolytic-uremic syndrome; VTEC, verotoxin-producing *Escherichia coli*.

2016) reporting the proportion of HUS cases associated with diarrhea. We assumed that all diarrhea-associated cases of HUS were caused by VTEC (Fakhouri *et al.*, 2017). All studies reported HUS cases in children, so we assumed that the proportion of cases due to VTEC was the same for adults. Supporting this assumption, VTEC-associated HUS is most common in young children and the elderly (Karpman *et al.*, 2017), and these two age groups made up the majority of HUS deaths and hospitalizations captured by our outcome data sources. For each disease, the best-fitting distribution for the set of literature-sourced point values was determined with @RISK. The appropriate proportion was then multiplied by the count of GBS or HUS outcomes for each year (2008–2012) to estimate how many were due to infections with *Campylobacter* or VTEC, respectively. The estimated proportions were 0.09–0.47 (uniform distribution) for *Campylobacter*-associated GBS and 0.75–1.00 (uniform distribution) for VTEC-associated HUS.

Nonspecific gastroenteritis

To capture deaths, hospitalizations, and ED visits due to nonspecific gastroenteritis, we used ICD-10/ICD-10-CA codes for both infectious and unspecified gastroenteritis (Thomas *et al.*, 2015). Nonspecific infectious gastroenteritis codes were as follows: A04.9 (bacterial intestinal infection, unspecified), A07.9 (protozoal intestinal disease, unspecified), A08.4 (viral intestinal infection, unspecified), A09 (diarrhea and gastroenteritis of presumed infectious origin), and A09.0 (other and unspecified gastroenteritis and colitis of infectious origin). Unspecified gastroenteritis codes were as follows: A09.9 (gastroenteritis and colitis of unspecified origin), K52.9 (noninfective gastroenteritis and colitis, unspecified), and R19.x (other symptoms and signs involving the digestive system and abdomen). For physician office visits, we captured unspecified gastroenteritis using the OHIP diagnostic code 009.

Before applying the AF for foodborne gastroenteritis, we estimated the proportion of unspecified gastroenteritis deaths and health care utilization due to pathogens or toxic substances (i.e., potential foodborne agents), as opposed to preexisting conditions. This proportion was based on four studies of self-reported acute gastrointestinal illness in Canadian communities (Majowicz *et al.*, 2004; Thomas *et al.*, 2006, 2017; Sargeant *et al.*, 2008). Each study reported the proportion of cases of recent vomiting or diarrhea due to preexisting conditions (e.g., irritable bowel syndrome, medication use, or pregnancy). The proportion was defined as a uniform distribution using @RISK, with its minimum and maximum values (0.81 and 0.88) corresponding to the smallest and largest of the four literature-sourced values. After applying the proportion, the counts of deaths and health care utilizations for unspecified gastroenteritis were added to the corresponding counts for infectious gastroenteritis. The total counts were then multiplied by the AF for foodborne gastroenteritis.

Further analytical details

For the DAD and NACRS data extracts, ~95% of hospitalizations and ED visits were assigned only one ICD-10-CA code for a disease included in our hazard-disease pairing list. For the ~5% of hospitalizations or ED visits with multiple relevant diagnostic codes, we considered the diagnosis type associated with each diagnostic code to decide what code best represented the utilization. If the most responsible diagnosis (DAD) or main problem (NACRS) was included, any other relevant diagnostic codes were excluded. The most responsible diagnosis was the diagnosis most responsible for a hospitalization (i.e., in terms of stay duration and use of resources), while the main problem was the clinically significant reason for an ED visit (and responsible for the greatest use of resources). If a most responsible diagnosis was not included among the multiple relevant diagnoses, we prioritized DAD diagnosis

types as follows: preadmit comorbidity, service transfer diagnosis, and postadmit comorbidity. If a main problem was not included, we considered all relevant other problems. If there were multiple diagnostic codes with the same diagnosis type, we selected one at random. Notably, there was one key exception to the above decision process. If the most responsible diagnosis was nonspecific gastroenteritis (infectious or unspecified) and the other relevant diagnostic codes were for diseases expected to be immediately associated with gastroenteritis, the most responsible diagnosis was excluded.

As a sensitivity analysis, we used two other decision processes to analyze DAD and NACRS data (hospitalizations and ED visits with multiple relevant diagnostic codes): (1) we considered only the most responsible diagnosis (i.e., main problem), and (2) we considered every relevant diagnostic code (regardless of its type) instead of selecting only one per hospitalization or ED visit. When the most responsible diagnosis alone or all diagnosis types were used to capture ED visits, the resulting total burden estimate did not change appreciably. However, considering only the most responsible diagnosis for hospitalizations resulted in a mean total burden estimate that was roughly half (53%) of the original estimate. This decrease was largely driven by the decrease in hospitalizations due to nonspecific gastroenteritis. An appreciable increase in hospitalizations was not observed when considering all diagnosis types. Our sensitivity analyses suggest that our approach of considering multiple diagnosis types (prioritizing multiple diagnosis types by expected relative contribution to a hospitalization or ED visit) instead of only the most responsible diagnosis allowed us to better capture instances where nonspecific gastroenteritis was an important contributor to a hospital stay.

For aggregate data extracted from the OHIP database, each row contained the count of visits for a distinct combination of diagnostic code, billing (fee) code, calendar year, specialty billed, sex, and age group. To estimate physician office visits from OHIP billing data, we developed a process based on algorithms used by the Institute for Clinical Evaluative Sciences, a provincially-funded research institute that hosts a robust collection of patient-level Ontario health data. We first excluded visits billed by health care providers other than physicians or nurse practitioners. We summed the count of visits assigned a billing code with an "A" or "W" prefix (these are typically for assessments or consultations that are not part of ED or nonemergency acute care hospital in-patient services), excluding any billing codes typically used in hospital settings, for certification or pronouncement of death, or special visit premium codes typically accompanied by an "A" code (thus avoiding double counting a visit). Finally, we subtracted the count of visits assigned special visit premium codes for hospital-based services, which are typically accompanied by an "A" code (thus not counting hospital visits assigned "A" codes).

The burden of each disease was reflected in the type and number of its outcomes (e.g., number of physician office visits, ED visits, hospitalizations, and deaths). For diseases due to specific pathogens, we did not exclude extraintestinal syndromes, provided they could result from exposure to food.

Age and Sex Analysis for Nonspecific Gastroenteritis

To check for differences in outcomes across age and sex, we conducted a Poisson regression analysis on the death and health care utilization counts by sex (male, female) and age group (0–4, 5–19, 20–69, >70 years).

Supplementary Table S3. Poisson Regression Analysis of Nonspecific Gastroenteritis Outcomes by Sex and Age Group

Outcome	*Parameter*[a]	*Estimate*	p[b]
Physician office visits	Sex = male	–0.1085	<0.0001
	Age group = 0–4 years	1.3703	<0.0001
	Age group = 5–19 years	–0.1115	<0.0001
	Age group = 20–69 years	–0.2665	<0.0001
ED visits	Sex = male	–0.2065	<0.0001
	Age group = 0–4 years	1.3002	<0.0001
	Age group = 5–19 years	–0.3433	<0.0001
	Age group = 20–69 years	–0.5604	<0.0001
Hospitalizations	Sex = male	–0.1871	<0.0001
	Age group = 0–4 years	–0.3626	<0.0001
	Age group = 5–19 years	–2.3044	<0.0001
	Age group = 20–69 years	–1.9606	<0.0001
Deaths	Sex = male	–0.2452	0.0011
	Age group = 0–19 years[c]	–4.9408	<0.0001
	Age group = 20–69 years	–3.8785	<0.0001

[a]Reference groups were sex = female and age group = >70 years.
[b]Wald chi-square test.
[c]For deaths, due to small cell counts, we combined the 0–4 years and 5–19 years age groups.
ED, emergency department.

For nonspecific gastroenteritis, which accounted for most of our estimated burden, more females than males died, were hospitalized, visited the ED, or visited a physician's office ($p < 0.005$ for all comparisons) (Supplementary Table S3). Death and hospitalization counts were higher for those aged >70 years compared to other age groups ($p < 0.005$ for all comparisons). ED visit and physician office visit counts were higher for those aged 0–4 compared to the >70 years age group, and the counts were higher for the >70 years age group compared to those aged 5–19 years and 20–69 years ($p < 0.005$ for each comparison).

Our finding that more females than males died or sought health care due to nonspecific gastroenteritis is consistent with survey-based studies reporting a higher prevalence of acute gastrointestinal illness in females than males in Ontario (Majowicz *et al.*, 2004; Sargeant *et al.*, 2008) and the United States (Jones *et al.*, 2007). We found higher ED visits and physician office visits due to nonspecific gastroenteritis in those aged 0–4 years compared to other age groups, in agreement with reports of higher prevalence of acute gastrointestinal illness in those aged <10 years in Ontario (Sargeant *et al.*, 2008) and in those aged <5 years in the United States (Jones *et al.*, 2007).

Supplementary References

Ardissino G, Salardi S, Colombo E, Testa S, Borsa-Ghiringhelli N, Paglialonga F, Paracchini V, Tel F, Possenti I, Belingheri M, Civitillo CF. Epidemiology of haemolytic uremic syndrome in children. Data from the North Italian HUS network. Eur J Pediatr 2016;175:465–473.

Butler AJ, Thomas MK, Pintar KD. Expert elicitation as a means to attribute 28 enteric pathogens to foodborne, waterborne, animal contact, and person-to-person transmission routes in Canada. Foodborne Pathog Dis 2015;12:335–344.

Canadian Institute for Health Information (CIHI). *CIHI Data Quality Study of Ontario Emergency Department Visits for 2004–2005: Volume II of IV—Main Study Findings*. Ottawa, Canada: CIHI, 2008.

Canadian Institute for Health Information (CIHI). *CIHI Data Quality Study of the DAD 2009–2010 Discharge Abstract Database*. Ottawa, Canada: CIHI, 2012.

Constantinescu AR, Bitzan M, Weiss LS, Christen E, Kaplan BS, Cnaan A, Trachtman H. Non-enteropathic hemolytic uremic syndrome: Causes and short-term course. Am J Kidney Dis 2004;43:976–982.

Drenthen J, Yuki N, Meulstee J, Maathuis EM, van Doorn PA, Visser GH, Blok JH, Jacobs BC. Guillain-Barré syndrome subtypes related to *Campylobacter* infection. J Neurol Neurosurg Psychiatry 2011;82:300–305.

Elliott EJ, Robins-Browne RM, O'loughlin EV, Bennett-Wood V, Bourke J, Henning P, Hogg GG, Knight J, Powell H, Redmond D. Nationwide study of haemolytic uraemic syndrome: Clinical, microbiological, and epidemiological features. Arch Dis Child 2001;85:125–131.

Espié E, Grimont F, Mariani-Kurkdjian P, Bouvet P, Haeghebaert S, Filliol I, Loirat C, Decludt B, Minh NN, Vaillant V, de Valk H. Surveillance of hemolytic uremic syndrome in children less than 15 years of age, a system to monitor O157 and non-O157 Shiga toxin-producing *Escherichia coli* infections in France, 1996–2006. Pediatr Infect Dis J 2008;27:595–601.

Fakhouri F, Zuber J, Frémeaux-Bacchi V, Loirat C. Haemolytic uraemic syndrome. Lancet 2017;390:681–696.

Gerber A, Karch H, Allerberger F, Verweyen HM, Zimmerhackl LB. Clinical course and the role of Shiga toxin-producing *Escherichia coli* infection in the hemolytic-uremic syndrome in pediatric patients, 1997–2000, in Germany and Austria: A prospective study. J Infect Dis 2002;186:493–500.

Guarino M, Casmiro M, D'Alessandro R, Ravasio A. *Campylobacter jejuni* infection and Guillain-Barré syndrome: A case-control study. Neuroepidemiology 1998;17:296–302.

Hadden RD, Karch H, Hartung HP, Zielasek J, Weissbrich B, Schubert J, Weishaupt A, Cornblath DR, Swan AV, Hughes RA, Toyka KV. Preceding infections, immune factors, and outcome in Guillain–Barré syndrome. Neurology 2001; 56:758–765.

Hoffmann S, Batz MB, Morris Jr JG. Annual cost of illness and quality-adjusted life year losses in the United States due to 14 foodborne pathogens. J Food Prot 2012;75:1292–1302.

Jacobs BC, Rothbarth PH, Van der Meché FG, Herbrink P, Schmitz PI, De Klerk MA, Van Doorn PA. The spectrum of antecedent infections in Guillain-Barre syndrome a case-control study. Neurology 1998;51:1110–1115.

Jenssen GR, Vold L, Hovland E, Bangstad HJ, Nygård K, Bjerre A. Clinical features, therapeutic interventions and long-term aspects of hemolytic-uremic syndrome in Norwegian children: A nationwide retrospective study from 1999–2008. BMC Infect Dis 2016;16:285.

Jones TF, McMillian MB, Scallan E, Frenzen PD, Cronquist AB, Thomas S, Angulo FJ. A population-based estimate of the substantial burden of diarrhoeal disease in the United States; FoodNet, 1996–2003. Epidemiol Infect 2007;135: 293–301.

Jung JK, Skinner K. Foodborne and waterborne illness among Canadian Indigenous populations: A scoping review. Can Commun Dis Rep 2017;43:7–13.

Karpman D, Loos S, Tati R, Arvidsson I. Haemolytic uraemic syndrome. J Intern Med 2017;281:123–148.

Keegan VA, Majowicz SE, Pearl DL, Marshall BJ, Sittler N, Knowles L, Wilson JB. Epidemiology of enteric disease in C-EnterNet's pilot site—Waterloo region, Ontario, 1990 to 2004. Can J Infect Dis Med Microbiol 2009;20:79–87.

Koga M, Ang CW, Yuki N, Jacobs BC, Herbrink P, Van der Meche FG, Hirata K, Van Doorn PA. Comparative study of preceding *Campylobacter jejuni* infection in Guillain-Barré syndrome in Japan and The Netherlands. J Neurol Neurosurg Psychiatry 2001;70:693–695.

Kwong JC, Crowcroft NS, Campitelli MA, Ratnasingham S, Daneman N, Deeks SL, Manuel DG; Ontario Burden of Infectious Disease Study Advisory Group. *Ontario Burden of Infectious Disease Study (ONBOIDS): An OAHPP/ICES report*. Toronto, Canada: Ontario Agency for Health Protection and Promotion, Institute for Clinical Evaluative Sciences, 2010.

Lukacsovics A, Nesbitt A, Marshall B, Asplin R, Stone J, Embree G, Hurst M, Pollari F. Using environmental health officers' opinions to inform the source attribution of enteric disease: Further analysis of the "most likely source of infection." BMC Public Health 2014;14:1258.

Lynn RM, O'Brien SJ, Taylor CM, Adak GK, Chart H, Cheasty T, Coia JE, Gillespie IA, Locking ME, Reilly WJ, Smith HR. Childhood hemolytic uremic syndrome, United Kingdom and Ireland. Emerg Infect Dis 2005;11:590–596.

Majowicz SE, Dore K, Flint JA, Edge VL, Read S, Buffett MC, McEwen S, McNab WB, Stacey D, Sockett P, Wilson JB. Magnitude and distribution of acute, self-reported gastrointestinal illness in a Canadian community. Epidemiol Infect 2004;132:607–617.

Morton VK, Thomas MK, McEwen SA. Estimated hospitalizations attributed to norovirus and rotavirus infection in Canada, 2006–2010. Epidemiol Infect 2015;143:3528–3537.

Ontario Agency for Health Protection and Promotion (Public Health Ontario). Reportable Disease Trends in Ontario. Toronto, Canada. 2017. Available at: www.publichealthontario.ca/en/dataandanalytics/pages/rdto.aspx, accessed October 1, 2017.

Ontario Ministry of Health and Long-Term Care. Appendix B: Provincial Case Definitions for Reportable Diseases. 2014. Available at: www.health.gov.on.ca/en/pro/programs/publichealth/oph_standards/docs/vtec_cd.pdf, accessed October 23, 2017.

Prüss-Ustün A, Wolf J, Corvalán C, Bos R, Neira M. *Preventing Disease Through Healthy Environments: A Global Assessment of the Burden of Disease from Environmental Risks*. Geneva, Switzerland: World Health Organization, 2016.

Public Health Agency of Canada. *National Enteric Surveillance Program (NESP) annual summary 2010*. Ottawa, Canada. 2012. Available at: http://publications.gc.ca/collections/collection_2012/aspc-phac/HP37-15-2010-eng.pdf, accessed January 19, 2018.

Sargeant JM, Majowicz SE, Snelgrove J. The burden of acute gastrointestinal illness in Ontario, Canada, 2005–2006. Epidemiol Infect 2008;136:451–460.

Sivadon-Tardy V, Porcher R, Orlikowski D, Ronco E, Gault E, Roussi J, Durand MC, Sharshar T, Annane D, Raphael JC, Megraud F. Increased incidence of *Campylobacter jejuni*-associated Guillain-Barré syndromes in the Greater Paris area. Epidemiol Infect 2014;142:1609–1613.

Thomas MK, Majowicz SE, MacDougall L, Sockett PN, Kovacs SJ, Fyfe M, Edge VL, Doré K, Flint JA, Henson S, Jones AQ. Population distribution and burden of acute gastrointestinal illness in British Columbia, Canada. BMC Public Health 2006;6:307.

Thomas MK, Murray R, Flockhart L, Pintar K, Fazil A, Nesbitt A, Marshall B, Tataryn J, Pollari F. Estimates of foodborne illness-related hospitalizations and deaths in Canada for 30 specified pathogens and unspecified agents. Foodborne Pathog Dis 2015;12:820–827.

Thomas MK, Murray R, Flockhart L, Pintar K, Pollari F, Fazil A, Nesbitt A, Marshall B. Estimates of the burden of foodborne illness in Canada for 30 specified pathogens and unspecified agents, circa 2006. Foodborne Pathog Dis 2013; 10:639–648.

Thomas MK, Murray R, Nesbitt A, Pollari F. The incidence of acute gastrointestinal illness in Canada, Foodbook survey 2014–2015. Can J Infect Dis Med Microbiol 2017;5956148.

Tozzi AE, Caprioli A, Minelli F, Gianviti A, De Petris L, Edefonti A, Montini G, Ferretti A, De Palo T, Gaido M, Rizzoni G. Shiga toxin-producing *Escherichia coli* infections associated with hemolytic uremic syndrome, Italy, 1988–2000. Emerg Infect Dis 2003;9:106–108.

Vrbova L, Johnson K, Whitfield Y, Middleton D. A descriptive study of reportable gastrointestinal illnesses in Ontario, Canada, from 2007 to 2009. BMC Public Health 2012; 12:970.

Whitfield Y, Johnson K, Hobbs L, Middleton D, Dhar B, Vrbova L. Descriptive study of enteric zoonoses in Ontario, Canada, from 2010–2012. BMC Public Health 2017; 17:217.

Zautner AE, Johann C, Strubel A, Busse C, Tareen AM, Masanta WO, Lugert R, Schmidt-Ott R, Groß U. Seroprevalence of campylobacteriosis and relevant post-infectious sequelae. Eur J Clin Microbiol Infect Dis 2014;33:1019–1027.
